# Point-of-care measurements reveal release of purines into venous blood of stroke patients

**DOI:** 10.1007/s11302-019-09647-4

**Published:** 2019-03-12

**Authors:** Nicholas Dale, Faming Tian, Ravjit Sagoo, Norman Phillips, Chris Imray, Christine Roffe

**Affiliations:** 1 School of Life Sciences, University of Warwick, Coventry, UK; 2 Sarissa Biomedical Ltd, Unit 4b Vanguard Centre, Sir William Lyons Road, Coventry, UK; 3 University Hospitals of Coventry and Warwickshire, Clifford Bridge Road, Coventry, UK; 4 Patient Representative, University Hospitals of Coventry and Warwickshire, Clifford Bridge Road, Coventry, UK; 5 Warwick Medical School, University of Warwick, Coventry, UK; 6 University Hospital of North Midlands, Stoke-on-Trent, and Keele University, Keele, UK

**Keywords:** Purines, Stroke, Cerebral ischemia, Biosensor, Point of care

## Abstract

Stroke is a leading cause of death and disability. Here, we examine whether point-of-care measurement of the purines, adenosine, inosine and hypoxanthine, which are downstream metabolites of ATP, has potential to assist the diagnosis of stroke. In a prospective observational study, patients who were suspected of having had a stroke, within 4.5 h of symptom onset and still displaying focal neurological symptoms at admission, were recruited. Clinical research staff in the Emergency Departments of two hospitals used a prototype biosensor array, SMARTCap, to measure the purines in the venous blood of stroke patients and healthy controls. In controls, the baseline purines were 7.1 ± (SD) 4.2 μM (*n* = 52), while in stroke patients, they were 11.6 ± 8.9 μM (*n* = 76). Using the National Institutes for Stoke Scale (NIHSS) to band the severity of stroke, we found that minor, moderate and severe strokes all gave significant elevation of blood purines above the controls. The purine levels fall over 24 h. This was most marked for patients with haemorrhagic strokes (5.1 ± 3.6 μM, *n* = 9 after 24 h). The purine levels measured on admission show a significant correlation with the volume of affected brain tissue determined by medical imaging in patients who had not received thrombolysis or mechanical thrombectomy.

ClinicalTrials.gov Identifier: NCT02308605

## Introduction

Throughout the world, stroke is one of the leading causes of death and disability [1–3]. In the USA, there are approximately 795,000 strokes per year, and it is estimated that someone has a stroke every 40 s [3]. About 87% of these strokes are ischaemic, 10% caused by intracerebral haemorrhage and 3% by subarachnoid haemorrhage [3]. For patients suffering an ischaemic stroke, thrombolytic treatment can greatly improve outcome. However, speed is of the essence as during an ischaemic stroke as brain tissue is lost at an alarmingly rapid rate; for example, the brain has been estimated to prematurely age by 3.1 weeks for every minute of an untreated stroke [4]. Rapid lysis of thrombi by administration of alteplase significantly improves recovery [5]. A detailed study has estimated that for every 15-min reduction of delays in provision of treatment, there is on average a gain of one extra month of disability-free life [6].

Evaluation of how quickly stroke units provide thrombolytic treatment after arrival at hospital suggests that although many centres around the world achieve delays of < 30 min, typical routine practice still leaves room for substantial improvement [6]. Furthermore, given the rate of loss of brain matter and the benefit of early thrombolysis, “fine-tuning of even the best of services is beneficial” [6].

Current thinking suggests that improving the door to needle time (DNT) for thrombolysis requires “continuous analysis and stepwise improvement of the system as a whole” [6]. Early recognition and diagnosis of a stroke is clearly a vital component for achieving rapid DNTs. While diagnosis of stroke will always require clinical examination and judgement, this process can be assisted and potentially sped up by analytical technologies. Use of proteomic biomarker panels has shown how, albeit imperfect, analytical tools can be integrated with other clinical data to assist in the early diagnosis and management of stroke patients [7, 8].

In this study, we examine whether point-of-care purine measurements have potential to assist in the diagnosis of stroke. The purines adenosine, inosine and hypoxanthine are downstream metabolites of ATP. In tissue that is under metabolic stress, e.g. during hypoxia or ischaemia, the intracellular ATP-pool breaks down to adenosine and downstream metabolites. These purines are released from cells via transporters and are thus a very early marker of metabolic stress that occurs prior to cell death [9–20]. Using the cross-clamp phase of carotid endarterectomy as a model of diffuse brain hypoxia/ischaemia, adenosine measured in blood has been shown to be a very sensitive marker of brain ischaemia [21].

Purine measurements have not been widely used in clinical research because they are technically difficult and labour-intensive to achieve. Once blood has been drawn from the patient, the purines are subject to a number of degradative and uptake processes that cause their half-life in blood to be very short (minutes). This necessitates complex pretreatment of blood samples as soon as they are obtained to remove the cells and inactivate degradative processes to try and preserve the purine content of the sample. These methods are complex and prone to variation, and not suited to widespread application in a clinical setting. A point-of-care system for measurement of purines in unprocessed blood would obviate these complications and usher in a new era where measurement of the purines could be a new analytical tool for clinicians. We invented electrochemical biosensors for adenosine and downstream purines initially as a lab tool [22–24]. We have now developed these biosensors into a very early prototype biosensor array, SMARTCap, which is capable of measuring purines in fresh unprocessed blood. In this study, clinical research staff used SMARTCap in the Emergency Departments of two hospitals to investigate whether the purines are elevated in the blood of stroke patients compared to healthy controls.

## Materials and methods

### Ethics

This trial was approved by the Regional Coventry and Warwickshire Research Ethics Committee (14/WM/1067) on 19th August 2014. It is registered as NCT02308605.

### Trial design

This was a prospective observational study of purine levels in patients admitted with a clinical diagnosis of acute stroke.

### Participants

**Fig. 1:**
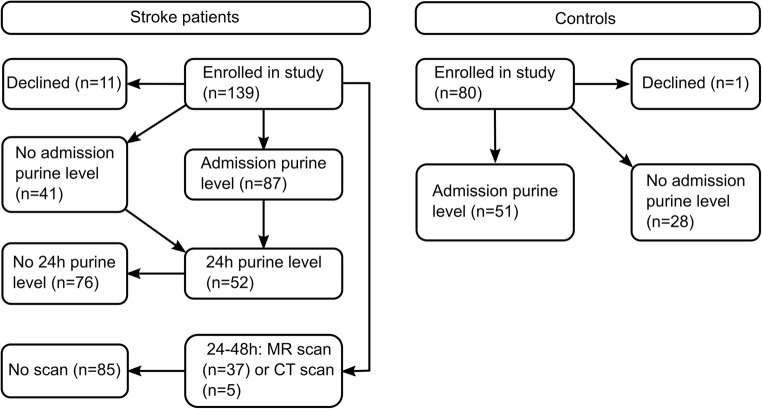
Consort diagram for the SMARTCap trial. Purine measurements on admission and at 24 h were not obtained because the measurement procedure ran into a problem and one of the steps could not be completed; or the measurement was completed but the resulting electrochemical data was spurious (see “Materials and methods”); or, for the 24-h purine level, the patient was repatriated before the measurement could be made. Twenty-four- to 48-h scans were not made because the MR scanner was not available; or the patient did not consent for an MR scan; or the patient was too ill for the MR scan; or the patient was repatriated before the scan could be made

Patients were recruited from the emergency departments of two large tertiary care hospitals (the University Hospital of Coventry and Warwick NHS Trust and the University Hospital of North Midlands NHS Trust) between 30 September 2014 and 10 July 2015 if they had a clinical diagnosis of suspected acute stroke e.g. Face Arm Speech Test (FAST) positive [25] or other relevant clinical signs [26], were within 4.5 h of symptom onset, and were still displaying focal neurological symptoms at the time of assessment. All participants entered the normal clinical care pathway for stroke at the respective hospital (Fig. 1).

### Controls

Healthy individuals of similar age were recruited as controls (Fig. 1). These were the relatives and friends of stroke patients recruited to the study. A brief questionnaire was given to exclude comorbid conditions and medications which might affect purine levels and to ensure that none of the healthy controls had been diagnosed as having vascular disease.

### Consent

As this was a trial in an emergency setting and informed consent would have delayed time critical investigations and treatment, we used a model of deferred consent. The first blood sample was taken as soon as possible after arrival in hospital, usually together with the routine emergency bloods. Fully informed consent was obtained from the patient, or, if the patient was incompetent to give consent, a legal representative as soon as practicable after emergency treatment and, before the next study-related activity (the 24-h blood sample). If consent could not be gained or was refused, no further trial procedures were undertaken and the result of the purine test conducted before consent and the data removed from the study (Fig. 1). Fully informed consent was obtained from all healthy individuals recruited as controls before commencement of any trial-related procedures.

### Blood sampling

As soon as possible after admission, blood from a consented patient (or a patient where retrospective consent was being sought) was drawn into a vacutainer and the SMARTCap device was used to measure purines in this sample. This first measurement was considered to be the admission or “0-h” measurement. Twenty-four hours later, the procedure was repeated.

### SMARTCap

**Fig. 2:**
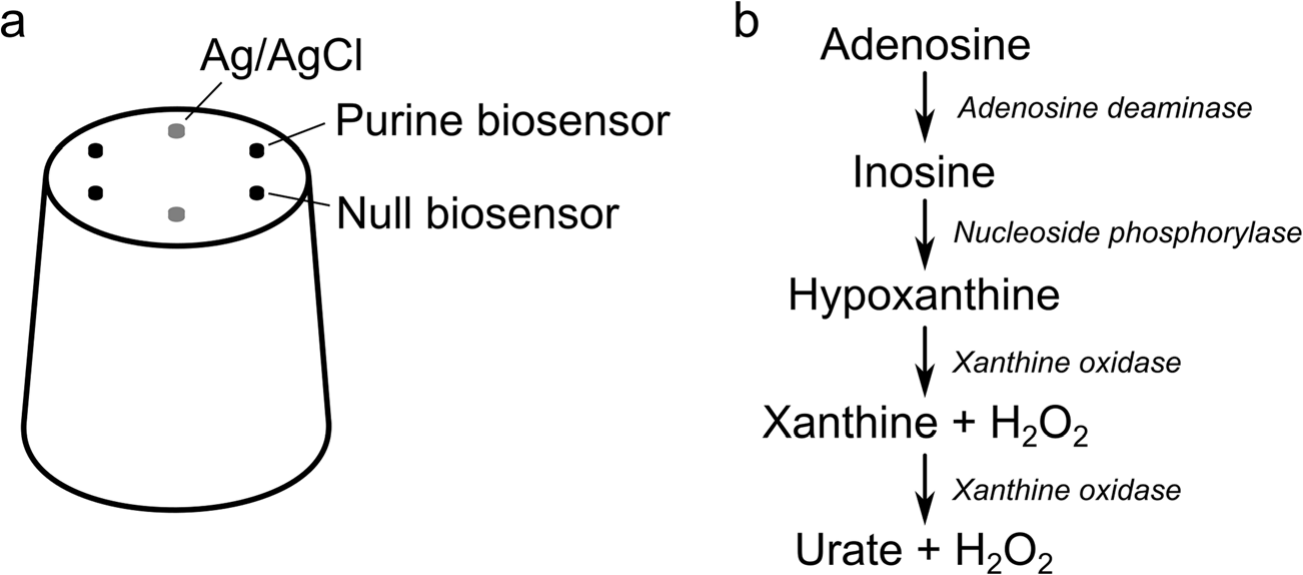
Design and operational principles of SMARTCap. **a** Diagram of SMARTCap showing the four working electrodes (black) and two Ag/AgCl pseudo-reference electrodes (grey). Two of the working electrodes were coated with a gel layer containing the enzymes for a purine biosensor. The other two working electrodes were coated with a gel layer lacking enzymes to comprise null biosensors). **b** Enzymatic cascade used on the working electrodes for detection of purines

The SMARTCap consisted of an array of four working electrodes and two Ag/AgCl pseudoreference electrodes (Fig. 2a). The working electrodes were gold and coated with ruthenium purple, which acts as a surface-bound mediator to create highly selective biosensors [24]. Two of the working electrodes were coated with the enzyme cascade required for purine detection (Fig. 2b), while the remaining two were coated with a layer that lacked the enzymes.

SMARTCap was used to make chronoamperometric recordings of the working electrodes at a potential of − 50 mV versus the Ag/AgCl reference electrode. When introduced into blood, the SMARTCap was polarised to its working potential of − 50 mV for 3 min. At the end of this period, the data values from the final 10 s of the recording were averaged for each working electrode. The purine biosensors are sensitive to adenosine, inosine, hypoxanthine and xanthine. The null sensors lack sensitivity to these compounds and act as a control for any non-specific interferences and also to set the “zero current” level for the absence of purines from the measurement sample. All measurements are thus performed as the difference between the two biosensor and two null electrodes giving a total of four measurements from one sample. These were averaged to give a single value.

The SMARTCap was designed to fit into a vacutainer to enable measurement of purines in fresh unprocessed blood. Prior to taking the blood sample, the SMARTCap was rehydrated and calibrated against a known concentration of adenosine. Once the blood was drawn into a vacutainer from the patient, the SMARTCap was introduced to the sample as quickly as possible and the measurement made.

The potentiostat that controlled the SMARTCap was driven by bespoke software, which gave instructions to the user at each stage and stored the electrochemical data. The purine measurement itself and data were not revealed to the nurses. Some quality checks were built into the software to check for faulty SMARTCaps, or faults in use. The nurses were notified if a measurement was successful or had failed.

### Validity of electrochemical data

The electrodes in SMARTCap follow well-understood electrochemical behaviour and exhibit faradaic charging curves when polarised to the working potential which start from a negative current and decay to a current value close to zero [24]. These charging curves were stored for subsequent inspection. For apparently successful measurements, i.e. they had passed the initial quality checks in the software, the recordings from any electrodes that did not exhibit the expected curves were rejected. Given the redundancy within SMARTCap (two nulls and two working electrodes), the calculation to determine the concentration of purines was performed with all valid charging curves. In the event that all of the charging curves were found to be invalid for a SMARTCap measurement, this was recorded as a failed measurement. For stroke patients, 24 measurements of purines on admission and 18 measurements at 24 h failed according to these criteria. For control patients, 21 measurements failed.

### Clinical assessments of patients

Demographic data, comorbidities, medications and the neurological status e.g. National Institutes for Health Stroke Scale (NIHSS) [27] were recorded at baseline. A purine measurement was taken as soon as possible after admission either before or immediately after the baseline computed tomography (CT) head scan that all participants had on admission as part of standard clinical care. The purine measurements were performed before any reperfusion treatment such as thrombolysis or thrombectomy had been commenced. At 24 h after enrolment, a second purine measurement was taken in participants who had given fully informed consent. Magnetic resonance imaging (MRI) of the head was conducted in consented participants within 24–48 h after enrolment to obtain imaging evidence to confirm the clinical diagnosis and give an estimate of the volume of affected brain tissue. In participants who had contraindications to MRI or were unable to tolerate the procedure, a CT scan was performed instead of the MRI.

### Image analysis and calculation of brain volume

A radiologist blinded to the patient’s details, presentation and purine measurements performed the image analysis. Brain CT scans undertaken at presentation and follow-up CT/MR scans undertaken at 24–48 h were imported into and analysed using OsiriX software (Pixmeo, Geneva, Switzerland). CT scans were performed unenhanced. The following brain MR sequences were utilised:

1. Standard sagittal T1, axial T2 and axial T2 fluid attenuated inversion recovery (T2-FLAIR)
2. Coronal susceptibility weighted imaging (SWI) to aid in identifying micro haemorrhages
3. Axial diffusion weighted imaging (DWI) with associated apparent diffusion coefficient (ADC) maps to confirm the presence of an acute infarction

A data collection sheet based on the Acute Ischaemic Stroke Classification Template developed by the Edinburgh Brain Research Imaging Centre was used to record all abnormalities identified on the CT and MR scans (SBIRC website, www.sbirc.ed.ac.uk/imageanalysis.html, J Wardlaw) [28, 29]. A diagnosis of acute infarction was made on MR if there was evidence of restricted diffusion on DWI/ADC as well as appropriate abnormalities seen in the remainder of the sequences. Acute infarctions were diagnosed on CT using well-known, classical radiological signs including loss of grey-white matter differentiation in a cerebral arterial territory (together with mass effect due to cerebral oedema) and a hyperdense major cerebral artery (usually the middle cerebral artery). CT scans were also utilised to assess for intracerebral haemorrhage.

Volumes of acute infarctions and intracerebral haemorrhages were calculated in OsiriX by manually tracing around the edges of the regions of interest on the DWI sequence and CT scan, respectively. This was done on each relevant image slice to obtain a volume via the in-built volume calculation tool.

### Statistical analysis

Inspection of the data showed that the controls, strokes and mimics appeared to be normally distributed. Therefore, parametric tests (ANOVA and unpaired *t* tests) were used to compare data from different groups. Given the scatter apparent in the data, correlations between variables were assessed with the Spearman rank correlation test. Receiver operating characteristic (ROC) analysis was performed with the aid of the pROC package in R.

### Funding

This project (II-LA-0313-20002) is funded by the National Institute for Health Research Invention for Innovation Programme. The views and opinions expressed therein are those of the authors and do not necessarily reflect those of the Invention for Innovation Programme, the National Institute for Health Research, the NHS or the Department of Health.

## Results

### Purine levels in stroke patients, stroke mimics, and controls

**Fig. 3:**
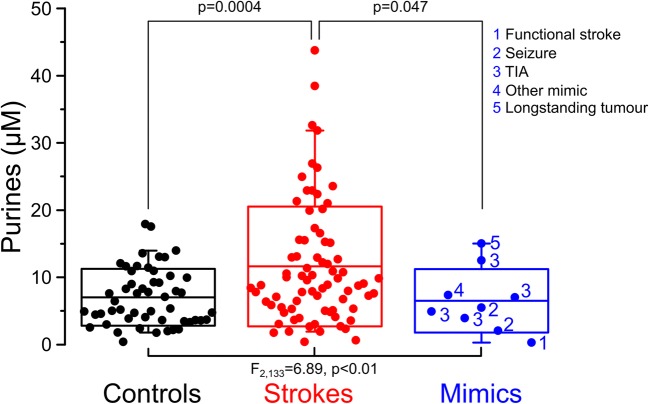
Purine levels in venous blood of stroke patients are elevated relative to those measured of healthy controls. Dot plots showing the values of purines measure with SMARTCap in healthy controls, stroke patients, and patients identified as stroke mimics. Box plots show mean and SD, whiskers are 5–95 percentiles. Comparison of three groups with three-level single factor ANOVA, followed by pairwise *t* tests

In healthy control patients, the purines measured in venous blood had a mean concentration of 7.1 ± (SD) 4.2 μM (*n* = 52, Fig. 3). In patients with a diagnosis of stroke, the mean purine concentration was 11.6 ± 8.9 μM (*n* = 76, Fig. 3). A small number of patients, initially diagnosed as strokes, were subsequently determined to be stroke mimics. The purine levels of these patients had a mean concentration of 6.5 ± 4.7 μM (*n* = 9) and are also illustrated in Fig. 3, along with the particular condition. Three further patients were not diagnosed as either a stroke or a stroke mimic: One of these suffered from cervical cord compression (admission purines 29.3 μM); one had venous sinus and internal jugular thrombosis (admission purines 15.9 μM); and the final patient had an unclear diagnosis, but myocardial infarction was suspected (admission purines 24.4 μM).

A three-level single-factor ANOVA suggested that the admission purine levels in controls, stroke patients and stroke mimics were significantly different (*F*_2.133_ = 6.89, *p* < 0.01). A separate post hoc comparison between stroke patients and controls showed that it was highly unlikely that they were drawn from the same underlying population (*p* = 0.0004). We therefore conclude that purines are elevated in the venous blood of stroke patients. In our patient sample, there were examples of both ischaemic and haemorrhagic strokes and the purine levels were elevated in both groups.

Although the numbers are too small to draw a confident conclusion, conditions that can be hard to distinguish from true strokes (functional strokes and seizures) seemed to have low purine levels (*p* = 0.047 compared to stroke). Individuals with TIAs exhibited a range of purine levels, perhaps reflecting the variability in severity of the TIA (Fig. 3).

### Purine levels versus the severity of stroke

**Fig. 4:**
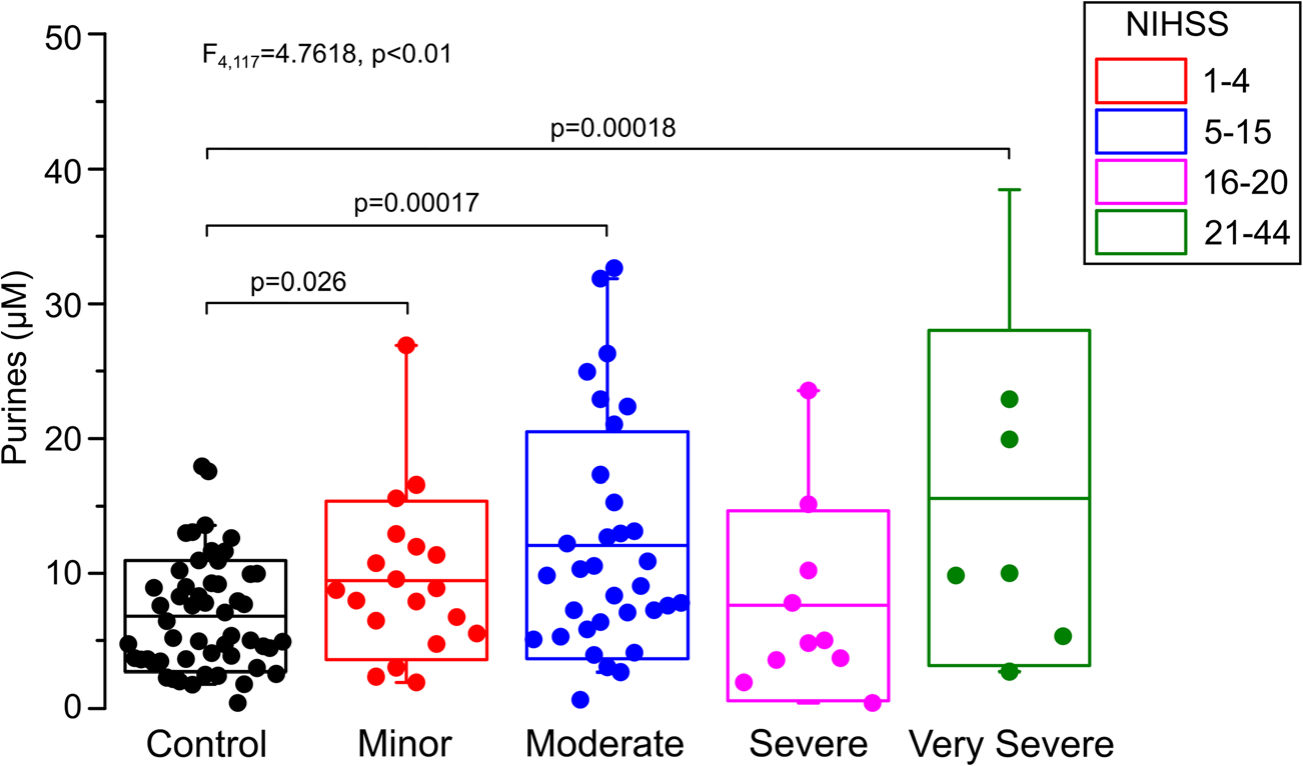
Purine levels measured in stroke patients grade with severity of stroke classified by NIHSS. A five-level single factor ANOVA shows that the purine levels in controls and stroke groups are significantly different. Pairwise *t* tests show that purine levels in minor, moderate and very severe stroke patients are significantly elevated relative to controls. Box plots show mean and SD, whiskers are 5–95 percentiles

As purines are released from cells that are metabolically stressed or that have died, the concentration of purines measured in blood might be expected to correlate with the severity of the stroke and the volume of affected brain tissue. We therefore used the baseline NIHSS to group patients into minor, moderate, severe and very severe strokes (Fig. 4). An analysis of variance, comparing these four bands along with the controls showed that the five groups were not drawn from the same population (*F*_4,117_ = 4.7618, *p* < 0.01). Subsequent post hoc comparisons showed that the minor, moderate and very severe stroke groups were all elevated with respect to the control. Overall, there was a progression that was suggesting that higher purine levels were associated with more severe strokes, but this correlation was not statistically significant (Fig. 4).

**Fig. 5:**
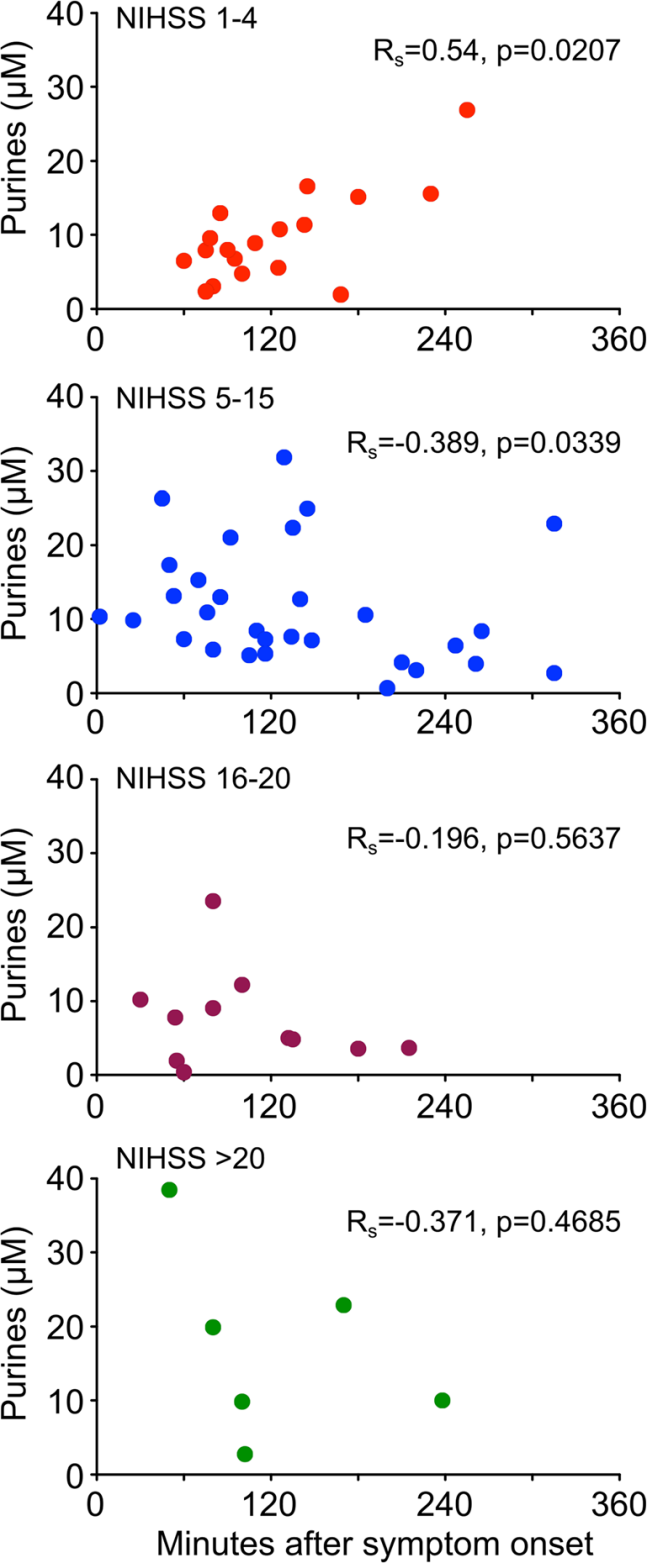
Purine levels in venous blood of stroke patients plotted against time from symptom onset, separated according to NIHSS. For minor strokes (NIHSS 1–4), there is a statistically significant positive correlation, i.e. the longer the delay in measurement, the higher the purine level in blood. For moderate strokes (NIHSS 5–15), there is a slight but statistically significant negative correlation, i.e. with symptom onset time to measurement delay, there is a slight fall in purine levels. With strokes of greater severity, a similar trend cannot be excluded, but the numbers are too small to interpret reliably. *R*_S_ Spearman’s rank correlation coefficient

One possible confounding factor in our study is that the measurements of purines in blood taken at admission were performed at differing times relative to the onset of symptoms. If the purines in blood were to rise slowly, or decay quickly, an influence of time of sampling on the measured purine level might be expected. Analysis of all the stroke patients showed no correlation between the time from symptom onset and the level of purines (*R*_s_ = 0.171). To examine this further, we categorised patients via their baseline NIHSS and to test whether there was any effect of measurement time relative to symptom onset on the measured purine levels in patients with strokes of differing severity (Fig. 5). Interestingly, there was a positive correlation (*R*_s_ = 0.54, *p* = 0.0207, Fig. 5) between time from stroke onset and venous purine levels for patients with minor strokes, but a negative correlation for patients with moderate strokes (*R*_s_ = − 0.389, *p* = 0.03389, Fig. 5). A similar trend was evident in patients with severe and very severe strokes; however, the correlation was not significant in these patients. This may have been due to the small number of patients in these more severe categories (Fig. 5).

### Purine levels versus affected brain volume

**Fig. 6:**
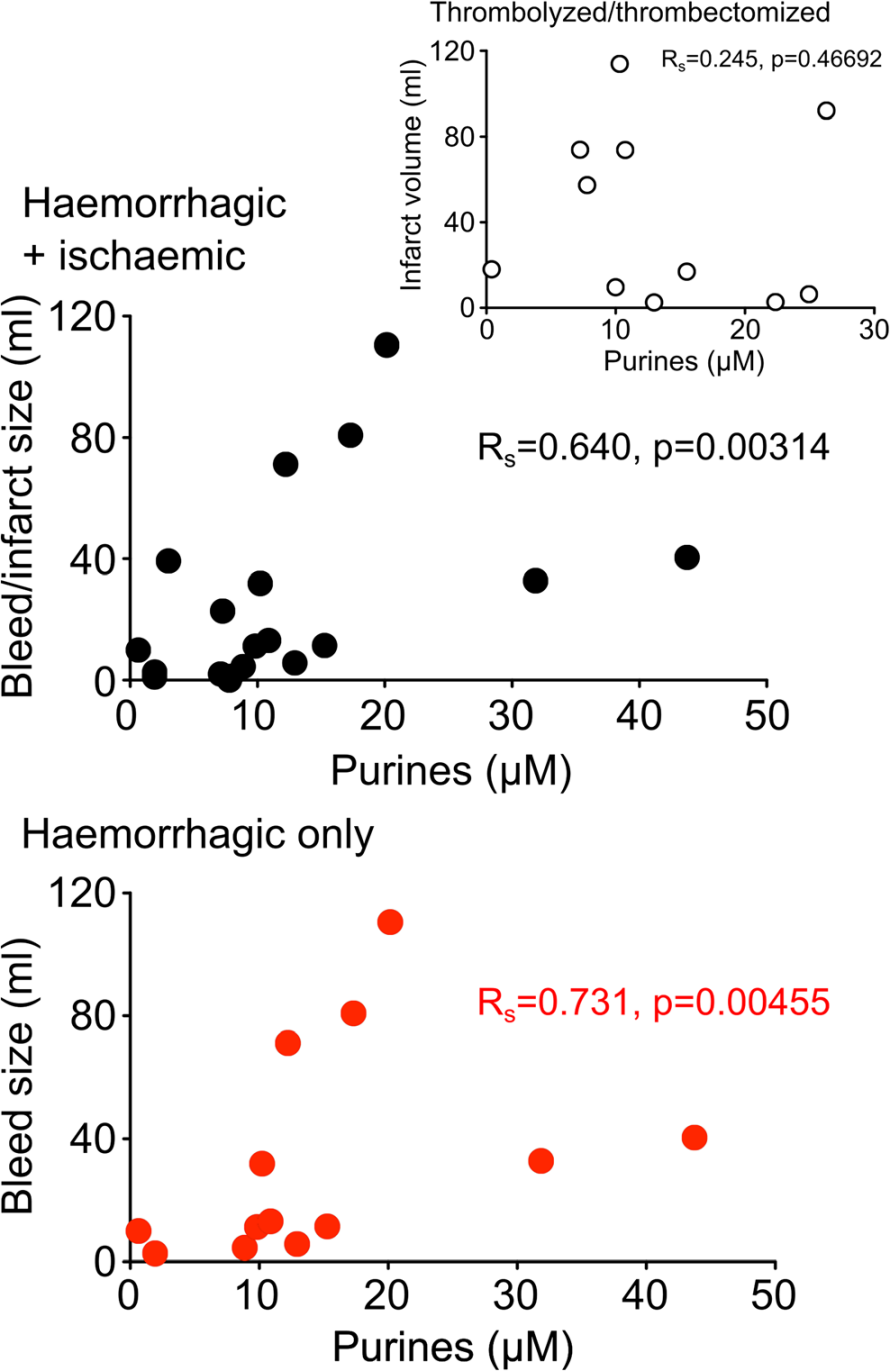
Purines measured at time of admission correlate with volume of affected brain tissue measured by CT or MR imaging. The top panel shows non-thrombolysed ischaemic and haemorrhagic patients; the bottom panel shows only haemorrhagic patients. The inset shows lack of correlation between purines measured on admission and infarct size measured by MRI in ischaemic stroke patients who had undergone thrombolysis/thrombectomy. CT scans performed the same day as admission for haemorrhagic patients, one ischemic stroke patient had CT scan performed at 24–48 h after admission, and the MRI scans performed 24–48 h after admission. *R*_S_ Spearman’s rank correlation coefficient

We performed medical imaging, either with CT on admission or MR 24–48 h after admission. This enabled us to test whether the purine levels measured *on admission* correlate with the volume of affected brain tissue evident in the imaging. In patients with a diagnosis of an ischaemic stroke and who had been given thrombolysis, no correlation between volume of affected brain (determined by MR scan) and the purine measurement was evident (Fig. 6, inset). This is reassuring, as the purpose of thrombolysis is to rescue the affected tissue. Although the region of affected brain will be highly stressed initially and thus release purines, the reperfusion arising from thrombolysis should prevent a significant proportion of these stressed cells from dying. Hence, we would expect the thrombolytic intervention to break any potential link between the initial purine levels and volume of affected brain.

We next examined patients who did not receive thrombolysis and plotted the initial purine levels in blood versus the volume of affected brain. A significant correlation between blood purine levels and volume of affected brain tissue was evident (*p* = 0.00314, Fig. 6). This group included both ischaemic and haemorrhagic strokes. When we analysed haemorrhagic strokes on their own, a correlation between purine levels and volume of affected brain determined by the CT scan on admission was also evident (*p* = 0.00455, Fig. 6). There was an insufficient number of non-thromoblysed ischaemic stroke patients in our dataset to warrant their separate analysis.

### Time course of purine levels over 24 h

In 36 stroke patients, a second purine measurement 24 h after admission was successfully completed. The mean purine level was 7.6 ± 6.5 μM. This value was significantly lower than that recorded on admission (*p* = 0.01) and was not significantly different from the control group, suggesting at least a partial return of purine levels towards those of healthy controls over the 24-h period.

**Fig. 7:**
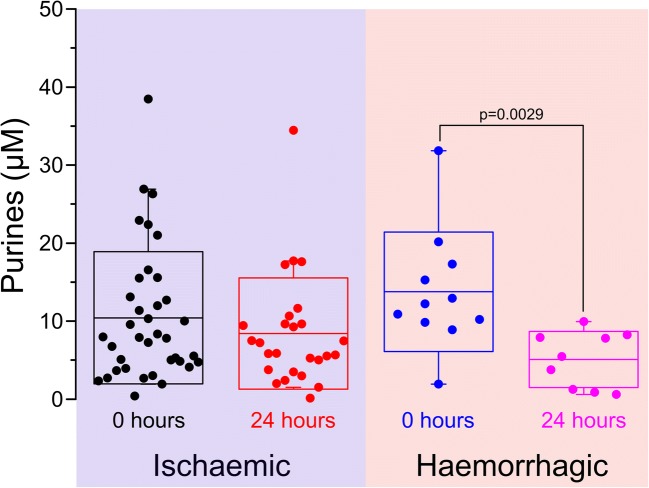
Purine levels show a trend to normalisation 24 h after admission. The purines measured at time of admission and at 24 h shown for ischaemic and haemorrhagic strokes. Comparison of 0 h and 24 h via *t* tests. The difference is not significant for ischaemic strokes

Further analysis of patients with a definite diagnosis of either ischaemic or haemorrhagic stroke suggested that the purine levels for ischaemic and haemorrhagic strokes recovered at different rates over 24 h (Fig. 7). When measured on admission, the mean purine level for ischaemic and haemorrhagic strokes was 11.0 ± 8.5 μM (*n* = 37) and 13.8 ± 7.7 μM (*n* = 11), respectively. After 24 h, the purine levels had respectively reduced to 8.4 ± 7.1 (*n* = 26) and 5.1 ± 3.6 μM (*n* = 9). This was not a significant change for the ischaemic stroke patients (*p* = 0.109); however, for the haemorrhagic stroke patients, this reduction of the purine levels over 24 h was highly significant (*p* = 0.0029).

## Discussion

The key findings of our study are that purines are elevated in the venous blood of stroke patients when compared to healthy controls and patients with conditions mimicking stroke. This rise in purines is found irrespective of the underlying cause of the stroke (infarct or haemorrhage) and can be identified in all levels of stroke severity. Within 24 h of stroke onset, purine levels decline and this is more marked in strokes caused by haemorrhage than in infarcts. In patients who did not receive thrombolysis, a treatment that has the potential to reverse cerebral ischaemia, there was a strong correlation between purine levels measured on admission and the volume of affected brain tissue. Although the sample numbers are low, our data suggests that the purine levels of at least some stroke mimics may be lower than those of true strokes. Purine measurements may have potential in identifying stroke mimics and distinguishing them from true stroke patients.

To our knowledge, this is the first point-of-care study to assess purine levels in the blood of stroke patients in comparison to healthy controls. An earlier study [30] examined adenosine levels in patients who had suffered either TIA (*n* = 10) or stroke (*n* = 10) by lab-based HPLC measurements. In this study, adenosine levels were claimed to be elevated above the normal range for several days. However, no age-matched controls were performed, the absolute levels of adenosine recorded were in the 100 s of nM, and the elevation above the presumed normal range very modest. In our study, we measured a combination of purines (adenosine, inosine and hypoxanthine), and consequently the absolute levels recorded were much higher, potentially giving a wider dynamic range for our measurements. By using a point of care in vitro diagnostic device, we have been able to sample a far larger number of patients and controls.

In patients with minor strokes as classified by their baseline NIHSS, there was a positive correlation between the time of measurement time relative to symptom onset time and the level of purines in venous blood. This could have been due to stroke progression. Should future studies confirm this hypothesis, repeated measurements of purine levels over the first few hours could have clinical utility in monitoring the development or resolution of the pathology in patients with strokes.

**Fig. 8:**
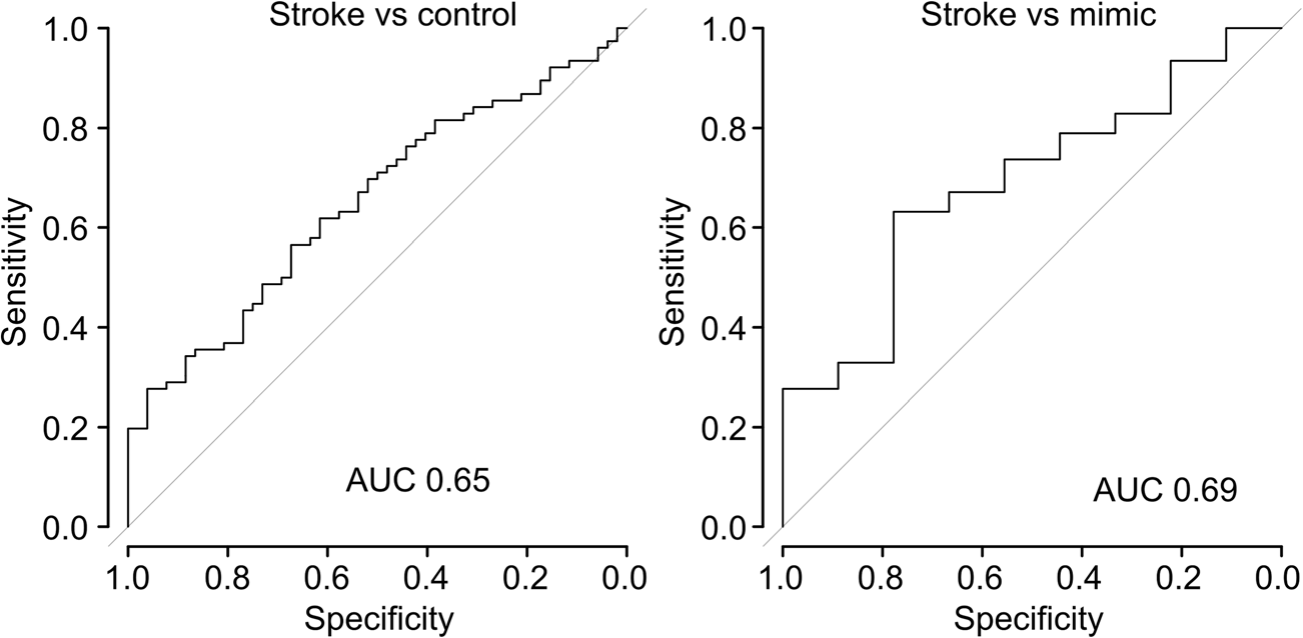
ROC analysis of venous purine levels in stroke patients versus healthy controls and mimics. The purines measured at time of admission show some discrimination of stroke versus controls and mimics, but the performance is not adequate for a diagnostic test. AUC area under the ROC curve

A limitation of this study is that variability in the measurements is higher than expected based on our previous observations and that the measured levels of purines in the healthy controls were some threefold greater than the literature would suggest. A priori, a level for purines in the low micromolar range with relatively little variance would be expected in controls. Indeed, in a previous study on patients undergoing carotid endarterectomy performed by researchers highly skilled in the use of biosensors (the inventors), the baseline purines measured in arterial blood were 2.4 ± 1.94 μM [31]. This suggests that systematic measurement/sampling errors may have affected our study. We think it unlikely that the values reflect a difference between arterial and venous blood, as recent studies using a next-generation device (SMARTChip) show very little difference in purine levels between finger-prick and arterial blood (O. Fisher, C. Imray, F. Tian, N. Dale unpublished observations). However, we are aware of three potential sources of error. Firstly, the SMARTCap was a very early prototype in vitro diagnostic device that was essentially handmade and developed specifically to enable this study. There was a high degree of variability between devices, and variation in their performance. We can state this with a high degree of certainty, because each SMARTCap had two purine biosensors and two null biosensors giving a total of four possible measurements. If the device were to work perfectly, all four measurements would be identical. Inspection of the raw data shows that this was never the case. This measurement variability arises from slight differences in the integrity of the coatings on the individual electrodes and hence their analytical performance. The final value for the purines was simply the mean of all of the valid (see “Materials and methods”) individual measurements. Secondly, SMARTCap, being a very early prototype device, proved difficult to use in the emergency setting. It took several training sessions, and experience through use, for the clinical research staff to attain proficiency in making measurements with SMARTCap. These difficulties resulted in a greater number of invalid measurements than originally expected. Furthermore, difficulties in use of SMARTCap may have led to delays in making the measurement of the blood samples. Metabolism, hypoxia-induced release or uptake of the purines into red blood cells in the ex vivo samples prior to completion of the measurement could have introduced further variability into the results. Thirdly, the purine measurements may have been affected by the method of blood draw. Obtaining the blood via a needle may lead to significant haemolysis and hence release of purines from damaged erythrocytes. These limitations affect the measurement of purines in the control group and the stroke group to the same extent. Despite these difficulties, use of SMARTCap was sufficient to enable the collection of the largest dataset of purine measurements from stroke patients and controls to date. Our study with SMARTCap demonstrates that the purines are indeed elevated in stroke patients relative to controls and that the level of purines correlates with the volume of affected brain tissue. To gain an idea of diagnostic performance, we performed a ROC analysis (Fig. 8) on the data to evaluate the discriminatory power of the SMARTCap test. For strokes versus controls, the area under the curve (AUC) was 0.65 (95% confidence interval 0.56 to 0.75), while for strokes versus mimics, the AUC was 0.68 (95% confidence interval 0.51 to 0.86). Clearly, this diagnostic performance is inadequate for clinical use. Nevertheless, given the technical limitations of SMARTCap and its use in the clinical setting, this performance is promising.

Our proof of principle study establishes that purines are released into the blood of stroke patients. Furthermore, this study confirms the finding of a related study in patients undergoing carotid artery surgery [31] that purines are produced within minutes of the onset of ischaemia. This could make purines better biomarkers for acute ischaemia than proteomic biomarkers, which require hours to become apparent. The purine levels detected in blood correlate with the volume of affected brain tissue determined by medical imaging. This in turn suggests that an early purine measurement has potential not only to help in identifying stroke patients but also to give information as to its severity that is independent of clinical assessment scales such as the NIHSS. Nevertheless, the present study is limited by the use of imperfect point-of-care measurement technology. As a result, the level of variance in the data precludes use as a diagnostic tool at this stage. However, our data warrant the development of better devices less prone to measurement error with better usability in a clinical and prehospital setting. The use of such devices will enable a more realistic evaluation of the diagnostic potential of purine release into blood for stroke that can be made.
